# Core genes in diverse dinoflagellate lineages include a wealth of conserved dark genes with unknown functions

**DOI:** 10.1038/s41598-018-35620-z

**Published:** 2018-11-21

**Authors:** Timothy G. Stephens, Mark A. Ragan, Debashish Bhattacharya, Cheong Xin Chan

**Affiliations:** 1 Institute for Molecular Bioscience, The University of Queensland, Brisbane, QLD 4072 Australia; 2 Department of Biochemistry and Microbiology, Rutgers University, New Brunswick, NJ 08901 USA; 3 School of Chemistry and Molecular Biosciences, The University of Queensland, Brisbane, QLD 4072 Australia

## Abstract

Dinoflagellates are a diverse group of unicellular primary producers and grazers that exhibit some of the most remarkable features known among eukaryotes. These include gigabase-sized nuclear genomes, permanently condensed chromosomes and highly reduced organelle DNA. However, the genetic inventory that allows dinoflagellates to thrive in diverse ecological niches is poorly characterised. Here we systematically assess the functional capacity of 3,368,684 predicted proteins from 47 transcriptome datasets spanning eight dinoflagellate orders. We find that 1,232,023 proteins do not share significant sequence similarity to known sequences, i.e. are “dark”. Of these, we consider 441,006 (13.1% of overall proteins) that are found in multiple taxa, or occur as alternative splice variants, to comprise the high-confidence dark proteins. Even with unknown function, 43.3% of these dark proteins can be annotated with conserved structural features using an exhaustive search against available data, validating their existence and importance. Furthermore, these dark proteins and their putative homologs are largely lineage-specific and recovered in multiple taxa. We also identified conserved functions in all dinoflagellates, and those specific to toxin-producing, symbiotic, and cold-adapted lineages. Our results demonstrate the remarkable divergence of gene functions in dinoflagellates, and provide a platform for investigations into the diversification of these ecologically important organisms.

## Introduction

Dinoflagellates are a diverse group of phytoplankton that are ubiquitous in marine and fresh waters. About 2300 dinoflagellate species have been described^1,2^, most of which are photosynthetic. However, mixotrophy^3,4^ that combines phototrophy and ingestion of prey (heterotrophy) is common. Photosynthetic dinoflagellates form the base of food webs and sustain global aquatic ecosystems via primary production and cycling of organic carbon and nitrogen. Bloom-forming dinoflagellates, predominantly in the orders Gonyaulacales and Gymnodiniales, can cause “red tides” (harmful algal blooms) and produce toxins that pose serious human health risks^5^. Other dinoflagellates, particularly Symbiodiniaceae^6^ (Suessiales), are symbionts in corals and other coral reef animals^7,8^. Dinoflagellates are also found in extreme environments, with multiple cold-adapted (psychrophilic) species described in the polar regions^9,10^. The capacity of dinoflagellates to thrive in diverse ecological niches, and the remarkable sequence divergence and complexity of their genomes when compared to other eukaryotes, have led researchers to grumble that dinoflagellates are in fact aliens from “outer space”^11^.

The genetic capacity and features that are common to all dinoflagellate lineages, or those related to niche specialisation (e.g., bloom formation, symbiotic lifestyle and cold adaptation), remain poorly understood. Symbiodiniaceae species are the only dinoflagellates for which genome data are available^12–15^. However, the functional capacity of dinoflagellate genes is poorly understood when relying on the commonly used annotation approach, whereby predicted proteins are compared against a set of curated proteins of known function that are largely derived from model organisms. The often-overlooked proteins of unknown function (i.e. “dark” proteins), and the corresponding dark genes, may be highly conserved in closely related species and represent unique lineage-specific features. Whereas genome data from dinoflagellates are limited, transcriptome data provide an avenue for the exploration of gene functions that drive niche specialisation in these species^16,17^.

Here we use available dinoflagellate transcriptome data to systematically investigate gene functions that are common (and unique) to distinct dinoflagellate lineages, and identify the conserved dark proteins. We also investigate gene functions and pathways that are enriched in toxin-producing, symbiotic, and cold-adapted dinoflagellates.

## Results and Discussion

We retrieved 64 publicly available dinoflagellate transcriptomes and their predicted proteins^18–20^ (Supplementary Table S1). To avoid potential biases arising from codon degeneracy, we restricted our analysis to proteins, using the amino acid sequences predicted from these transcriptomes. We filtered the datasets using stringent cri-teria, including the recovery of core conserved eukaryote proteins^21^ as an indicator of dataset completeness (see Methods). This approach resulted in the final 47 datasets, representing 3,368,684 protein sequences from eight taxonomic orders (Table 1).

**Table 1:** The final 47 datasets used in this study.

Taxon	Order	No. non-redundant protein sequences
*Dinophysis acuminata* DAEP01	Dinophysiales	83,934
*Alexandrium catenella* OF101	Gonyaulacales	68,889
*Alexandrium margalefi* AMGDE01CS-322	Gonyaulacales	50,502
*Alexandrium monilatum* CCMP3105	Gonyaulacales	87,380
*Alexandrium tamarense* CCMP1771	Gonyaulacales	114,975
*Azadinium spinosum* 3D9	Gonyaulacales	70,040
*Ceratium fusus* PA161109	Gonyaulacales	68,969
*Gambierdiscus australes* CAWD 149	Gonyaulacales	48,770
*Gambierdiscus caribaeus*	Gonyaulacales	290,362
*Gonyaulax spinifera* CCMP409	Gonyaulacales	39,652
*Lingulodinium polyedra* CCMP1738	Gonyaulacales	96,319
*Protoceratium reticulatum* CCCM535-CCMP1889	Gonyaulacales	75,595
*Pyrodinium bahamense* pbaha01	Gonyaulacales	99,554
*Amphidinium carterae* CCMP1314	Gymnodiniales	35,832
*Amphidinium massartii* CS-259	Gymnodiniales	49,240
*Gymnodinium catenatum* GC744	Gymnodiniales	82,846
*Karenia brevis* CCMP2229	Gymnodiniales	79,497
*Karenia brevis* SP1	Gymnodiniales	83,816
*Karenia brevis* SP3	Gymnodiniales	69,522
*Karenia brevis* Wilson	Gymnodiniales	90,529
*Karlodinium micrum* CCMP2283	Gymnodiniales	57,487
*Togula jolla* CCCM725	Gymnodiniales	42,196
*Noctiluca scintillans*	Noctilucales	40,801
*Oxyrrhis marina* LB1974	Oxyrrhinales	34,348
*Oxyrrhis marina*	Oxyrrhinales	43,246
*Brandtodinium nutricula* RCC3387 (“*Brandtodinium nutriculum*” in MMETSP)	Peridiniales	66,253
*Durinskia baltica* CSIRO CS-38	Peridiniales	88,656
*Glenodinium foliaceum* CCAP1116/3	Peridiniales	106,311
*Heterocapsa arctica* CCMP445	Peridiniales	45,573
*Heterocapsa rotundata* SCCAP K-0483	Peridiniales	43,925
*Heterocapsa triquestra* CCMP448	Peridiniales	57,688
*Kryptoperidinium foliaceum* CCMP1326	Peridiniales	161,360
*Peridinium aciculiferum* PAER-2	Peridiniales	53,784
*Scrippsiella hangoei*-like SHHI-4	Peridiniales	74,092
*Scrippsiella hangoei* SHTV5	Peridiniales	74,862
*Scrippsiella trochoidea* CCMP3099	Peridiniales	101,032
*Prorocentrum minimum* CCMP1329	Prorocentrales	85,555
*Prorocentrum minimum* CCMP2233	Prorocentrales	79,005
*Pelagodinium beii* RCC1491	Suessiales	47,797
*Polarella glacialis* CCMP1383	Suessiales	58,545
*Polarella glacialis* CCMP2088	Suessiales	33,576
*Symbiodinium* sp. C15 (*Cladocopium*)	Suessiales	37,221
*Symbiodinium* sp. C1 (*Cladocopium*)	Suessiales	45,710
*Symbiodinium* sp. CCMP2430 (*Symbiodinium*)	Suessiales	43,277
*Symbiodinium* sp. CCMP421 (*Effrenium*)	Suessiales	72,087
*Symbiodinium* sp. D1a (*Durusdinium*)	Suessiales	44,936
*“Symbiodinium”* sp. Mp	Suessiales	43,138

### Reference phylogeny and data completeness

**Figure 1:**
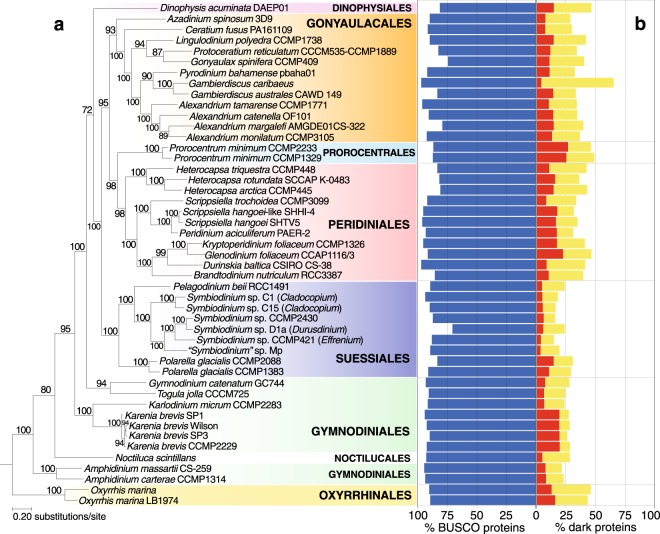
(**a**) Maximum-likelihood phylogeny inferred using the 1043 orthologous protein sets. Support values, based on 2000 ultrafast bootstrap approximations^24^, are shown at the internal nodes. The unit of branch length is the number of substitutions per site. (**b**) The percentage of recovered alveolate + stramenopile BUSCO proteins and of dark proteins in each dataset. High- and low-confidence dark proteins are shown in red and yellow bars, respectively.

An earlier study by Price and Bhattacharya^22^ demonstrated the utility of constructing a phylogeny using high-throughput transcriptome data. Following a similar approach^22^, we inferred a maximum-likelihood tree using these data comprising 1043 single-copy protein sets (Fig. 1a; see Methods). The statistics of the concatenated alignment (209,857 aligned positions) and the associated individual 1043 alignments used for inferring this tree are shown in Supplementary Tables S2 and S3, respectively. On average, each taxon contributes 22.13% of the aligned residues in the concatenated alignment (Supplementary Fig. S1A). The maximum-likelihood tree inferred from these sets (Fig. 1a) is largely topologically congruent to the published phylogeny^22^ (normalised Robinson-Foulds^23^ distance = 0.17). The backbone node for each taxonomic order is strongly supported (bootstrap support [BS] > 95% based on ultrafast bootstrap approximation^24^) in the tree (Fig. 1a) except for the Gonyaulacales and Gymnodiniales, as was also found in the earlier study^22^. Thus, phylogenetic signal from dinoflagellate transcriptomes is largely consistent in these two independent analyses. The sole member of the Dinophysiales, *Dinophysis acuminata* DAEP01, is placed as the basal lineage in the clade including Gonyaulacales, Prorocentrales, Peridiniales, and Suessiales (BS 72%; Fig. 1a); this taxon was sister to *Prorocentrum minimum* in the earlier published trees^22,25^. The placement of Dinophysiales at the base of this clade of five orders lends support to the earlier phylogeny and the single origin of the theca in dinoflagellates (comparable BS 72% in the tree of Janouškovec *et al*.^25^). The differential placement of Gonyaulacales and Suessiales relative to Peridiniales within this clade may be due to more aligned positions used for inferring the tree in Fig. 1a (based on 209,857 positions across 1043 protein sets) than those used in the earlier study^25^ (based on 29,400 positions across 101 protein sets). We note with caution that the high percentage of undetermined characters (on average 77.87% per taxon; Supplementary Table S2 and Fig. S1A) in our concatenated alignment may have resulted in a reduced information content, but 22.13% of this alignment, based on a larger number of protein sets, still comprises 46,449 amino acid positions. Although we required that each orthologous set contains sequences from ten or more taxa (see Methods), we cannot exclude the possibility that some sequences may have arisen from eukaryote prey of the mixotrophic taxa. However, the strong node support for each dinoflagellate order in the tree suggests that the impact of eukaryote contaminants on our inferred phylogeny is likely to be negligible. The presence of highly diverged homologs originating from non-dinoflagellate eukaryotic contaminants would likely weaken node support in the tree.

On average, 208.6 (89.1%) of the 234 alveolate + stramenopile BUSCO proteins^26^ were recovered in each of these 47 datasets, indicating their high extent of completeness (Fig. 1b). In an independent assessment at the order level (Supplementary Table S1), we recovered a high proportion of these conserved proteins, e.g. 233 of the 234 (99.6%) among the Peridiniales datasets. The sole dataset (*Dinophysis acuminata*) from the order Dinophysiales is reasonably complete, with the recovery of 190 (81.2% of 234) alveolate + stramenopile BUSCO proteins. The recovery of multiple homologs in some of the taxa may be due to true gene duplications or alternatively, reflect alternative splicing events.

### Prevalence of dark genes in dinoflagellates

Of all 3,368,684 proteins, 1,232,023 (36.57%) do not share significant sequence similarity to UniProt entries. The functions of these proteins are thus unknown, and we consider them as “dark” proteins. The average percentage of dark proteins in each dataset is 33%; the minimum is 15.2% in *Symbiodinium* sp. CCMP421 (now *Effrenium*), and the maximum is 63.5% in *Gambierdiscus caribaeus* (Fig. 1b). Although the number of dark proteins identified here may be somewhat dependent on the amount of data and the sequence length (low regression *R*^2^ values < 0.40 in Supplementary Fig. S2), these aspects have minimal impact on our broader interpretation that dark proteins are common in dinoflagellates.

We clustered the 3,368,684 protein sequences into 162,126 homologous sets of two or more sequences (see Methods). Of these sets (containing 2,554,321 proteins), 103,620 (63.9%) containing 441,006 proteins (17.27% of 2,554,321) are dark (hereafter the high-confidence set; see Methods). Within the 103,620 sets, 100,661 (97.14%) contain proteins from multiple taxa, whereas 2959 (2.86%) are taxon-specific; the latter must reflect e.g. alternative splice variants, because our approach excluded identical proteins from each taxon (Methods). The dark protein sets have an average size of 4.26, compared to the average size of 36.12 for the annotated sets, indicating that the dark protein families, although relatively more abundant, are smaller in size and more taxon-specific than annotated proteins. Of the 814,363 (unclustered) singleton proteins, 791,017 (97.13%) are dark (hereafter, the low-confidence set). These results suggest that dark proteins are prevalent in dinoflagellates and comprise an unexplored resource from which we can derive insights into the functional capabilities of these organisms.

### Are dark genes in dinoflagellates from outer space?

In the absence of functional annotation based on full-length protein sequences, conserved structural features such as protein domains can be used to illuminate the potential roles dark proteins play in dinoflagellate biology. The amino acid profile of the high-confidence dark proteins is largely similar to that of the annotated proteins; the proportions of four of the 20 amino acids are significantly different between the two sets (at 95% confidence interval of 10,000 comparisons of random subsamples; see Methods and Supplementary Fig. S3).

The putative functions of high-confidence dark proteins were further inferred though annotation of Pfam domains. Of the 441,006 proteins, only 6168 (1.4%) had Pfam annotations. In comparison, 31.38% of all proteins in this study were annotated with Pfam domains, indicating that these dark proteins are so highly diverged that their homologs (if any exist) are poorly represented in the curated databases. Although 202 (3.3%) of the 6168 Pfam-annotated dark proteins share significant similarity (BLASTP, *E* *≤* 10^−5^) with sequences in the more-inclusive RefSeq protein database, the majority (78.2%) of recovered top hits (Supplementary Table S4) are “hypothetical”, “uncharacterized”, “predicted”, “X-containing”, “X-like” or putative proteins. We therefore maintain that these proteins are dark. The dark proteins are shorter than the average length in these datasets (234.2 and 109.3 amino acids respectively for high- and low-confidence dark proteins, compared to 291.8 overall). This is likely not in itself sufficient to explain the inability to annotate dark proteins with functions^27^. It is possible (indeed likely) that some low-confidence dark proteins are artefacts arising from sequencing error or transcriptome mis-assembly. Of the 103,620 dark homologous sets, most (100,661; 97.14%) have proteins from multiple taxa. The recovery of these proteins in multiple datasets suggests that their prominence in dinoflagellates is unlikely to have arisen primarily from artefacts.

**Figure 2:**
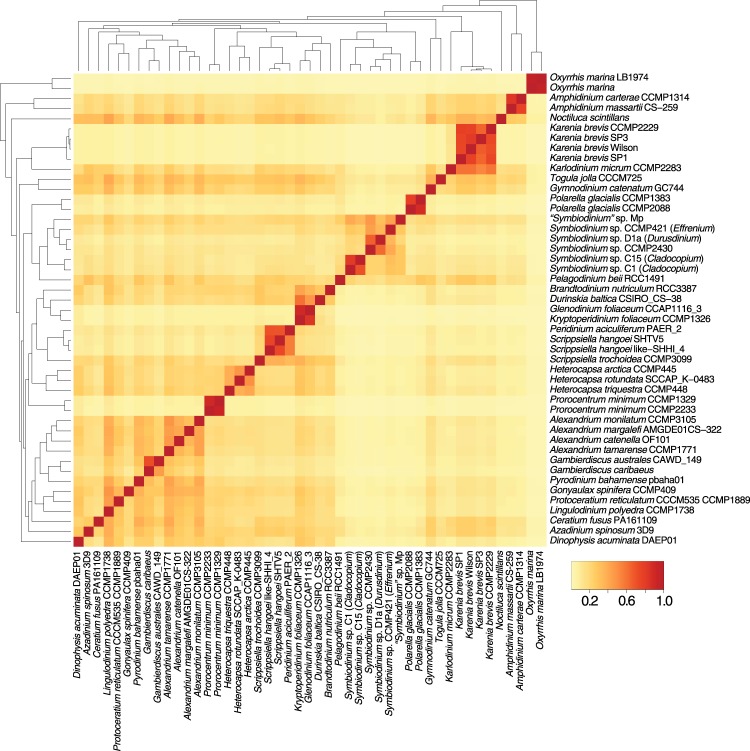
Heat map showing the proportion of dark protein sets shared between taxa used in this study. Each row is normalised by the total number of protein sets of which the taxon is a member. The order of the species on both axes and their associated dendrograms follow the phylogeny in Fig. 1a.

**Figure 3:**
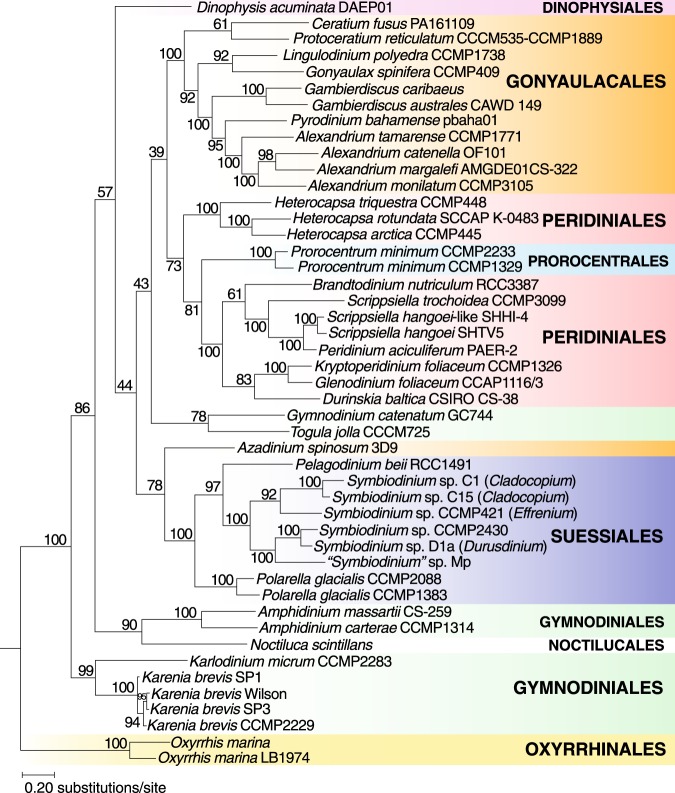
Maximum-likelihood phylogeny reconstructed using the 403 strictly orthologous dark protein sets. Support values, based on 2000 ultrafast bootstrap approximations^24^, are shown at the internal nodes. The unit of branch length is the number of substitutions per site.

Figure 2 shows the proportion of 100,661 multi-taxon dark protein sets that are shared pairwise between taxa, with reference to the phylogenetic relationship of these taxa (based on Fig. 1a). We observed higher proportions of these sets among closely related taxa, such as among the strains of *Karenia*, *Oxyrrhis*, and *Polarella*, indicating that these dark proteins are lineage- or species-specific innovations. Interestingly, 403/1043 (38.6%) of the single-copy sets used to construct our reference phylogenetic tree (Fig. 1a) are dark. The maximum-likelihood tree inferred from these 403 dark protein sets is shown in Fig. 3. The statistics of the concatenated alignment (71,346 aligned positions) are shown in Supplementary Table S5. Each taxon on average contributes to 18.98% of the aligned residues in the concatenated alignment (Supplementary Fig. S1B). The tree topology is largely congruent with our reference phylogeny in Fig. 1a, indicating that these dark proteins and dark protein sets are indeed dinoflagellate proteins (and unlikely to be artefacts), are predominantly lineage-specific, and are more rarely shared between distantly related lineages. This latter observation suggests a more general insight. Shared phylogenetic information is lost with time and divergence, supporting the adage that adaptive evolution is local^28^ and its footprints (be it novel gene origin or lateral genetic transfer) are most obvious in recently split taxa. The use of BUSCO proteins is useful for assessing genome completeness or broad patterns of genome growth/reduction but provides little insight into how specialised functions or lineages evolve. This is the realm of dark proteins that still remain poorly characterised.

Enrichment analysis comparing the annotated Pfam domains in high-confidence dark proteins and those in all datasets shows that functions related to calcium binding, protein localisation, protein degradation, protein-protein interaction, cell cycle regulation, and photosynthesis are over-represented (Supplementary Table S6). These functions may play a role in the ability of dinoflagellates to adapt to a rapidly changing environment, and may represent the putative functions of dark proteins. Protein degradation could be important for removing misfolded proteins that result from rapid changes in the local environment.

To further explore the conserved structural features of high-confidence dark proteins, we expanded our annotation strategy to include multiple methods and databases available via InterProScan. Using this approach, conserved features were annotated in 190,950 (43.3% of 441,006) high-confidence dark proteins. Of these, 37,270 proteins contain putative transmembrane domains (see Methods). We annotated conserved features in 36,352 proteins using SUPERFAMILY, ProSiteProfile, Pfam, PANTHER and Gene3D (in comparison to 6168 using Pfam alone). The ten most abundant domains identified by each of these in silico approaches are shown in Supplementary Table S7. The EF-hand, ubiquitin, zinc finger and IQ (calmodulin-binding) motifs are among the most abundant domains. The remaining 121,373 dark proteins are annotated with one or more secondary structures. Therefore, even though the functions of most dark proteins remain elusive, a substantial proportion of these proteins contain conserved structural features.

### Core functions in dinoflagellate lineages

For all proteins in each dataset, we annotated function based on significant sequence similarity to known proteins in UniProt, protein domains in Pfam^29^, membrane transporters^30^, and Gene Ontology terms (Supplementary Table S1). To assess the core protein functions in dinoflagellates, we identified the Pfam domains and membrane transporters that are the most abundant across all taxa (Supplementary Fig. S4). The prevalent domains and transporters that were recovered among the top ten and top 20 in each taxon are shown in Table 2. The prevalence of protein kinase, RNA recognition and ankyrin repeat domains implicates functions in a diverse array of important cellular processes, including proliferation, cell cycle, signal transduction and RNA splicing. The prevalent membrane transporters (Table 2) include those related to transport of ions, metabolites, sugars and lipids is critical to all dinoflagellates (i.e., as in most mixotrophic lineages), potentially for nutrient uptake and osmoregulation.

**Table 2:** Prevalent protein domains and membrane transporters annotated in dinoflagellate proteins consistently recovered among the top ten and among the top 20 in each of the 47 taxa.

Pfam domain (Pfam identifier)	Membrane transporter (family identifier)
Among top 10 in each taxon	
Protein kinase (PF00069)	Eukaryotic Nuclear Pore Complex (E-NPC) Family (1.I.1)
RNA recognition motif (PF00076)	Mitochondrial Carrier (MC) Family (2.A.29)
Ankyrin repeats (3 copies) (PF12796)	Ankyrin (Ankyrin) Family (8.A.28)
EF-hand domain pair (PF13499)	ATP-binding Cassette (ABC) Superfamily (3.A.1)
	Drug/Metabolite Transporter (DMT) Superfamily (2.A.7)
Among top 20 in each taxon	
WD40 repeat (PF00400)	Voltage-gated Ion Channel (VIC) Superfamily (1.A.1)
MORN repeat (PF02493)	The Major Facilitator Superfamily (MFS) (2.A.1)
	P-type ATPase (P-ATPase) Superfamily (3.A.3)

### Core functions in toxic dinoflagellates

To identify functions common to toxic dinoflagellates, protein annotations among taxa from Gonyaulacales and Gymnodiniales (hereinafter, the G + G dataset) were contrasted to those of all taxa. We found significant over-representations of the *Voltage-gated Ion Channel (VIC) Superfamily* (1.A.1) and the *Monovalent Cation:Proton Antiporter-1 (CPA1) Family* (2.A.36) in the G + G dataset (Supplementary Table S8).

The *Voltage-gated Ion Channel (VIC) Superfamily* (1.A.1) is the most over-represented membrane transporter family. These ion channels are critical in the maintenance of ion concentrations and gradients across cell membranes. The sodium and calcium voltage-gated ion channels are also the target for the majority of dinoflagellate toxins^31^. In eukaryotes, these channels are highly glycosylated with sialic acid, which is known to modulate the excitability of voltage-gated ion channels^32,33^. Pfam domains of *Glycosyltransferase family 29* (PF00777) and *Kelch motif* (PF01344), as well as the GO terms *sialylation* (GO:0097503) and *sialyltransferase activity* (GO:0008373) are over-represented in the G + G dataset. This indicates that functions related to the processing and attachment of sialic acids to other macromolecules are prominent in toxic dinoflagellates.

Whereas sialic acid had not been described in dinoflagellates^34^, it has been reported in other algae^35,36^. A gene related to sialyltransferase is differentially (more highly) expressed in toxin-producing strains of *Alexandrium minutum* than in non-toxic species^37^. Sialic acid was previously reported to be absent from the symbiotic dinoflagellates^38^. Here we found that the glycosyltransferase domain was almost completely absent from Symbiodiniaceae taxa (and from all lineages of Suessiales, except for three domain matches found in the *Symbiodinium* sp. CCMP421 [*Effrenium*] dataset).

Because voltage-gated ion channels are important in toxic dinoflagellates, the function of the channels must be unaffected by the toxins that these dinoflagellates release. In snakes, voltage-gated sodium channels that are resistant to tetrodotoxin (a toxin similar to saxitoxin from dinoflagellates^39^) have a reduced channel activity compared to those that are susceptible^40^. We hypothesise that a similar situation may occur in toxic dinoflagellates, i.e. voltage-gated ion channels are resistant to their own toxins and have a reduced activity. The link between sialic acid and these ion channels may represent a functional innovation in toxin-producing dinoflagellates, with the dinoflagellates using sialic acid to modulate (increase or recover) the activity of these toxin-resistant channels.

Known dinoflagellate toxins are polyketides produced by the multi-domain polyketide synthase (PKS) enzyme family^5^. The *Beta-ketoacyl synthase, N-terminal domain* (PF00109), one of the main PKS domains, and the *Beta-ketoacyl synthase, C-terminal domain* (PF02801) that is often associated with the N-terminal domain, are over-represented in the G + G dataset (Supplementary Table S8). The *Acyl transferase domain* (PF00698), another primary PKS domain, is over-represented with an adjusted *p*-value of 2.24 × 10^−6^. The cellular component GO term for *polyketide synthase complex* (GO:0034081) is also enriched.

### Core functions in symbiotic dinoflagellates

Dinoflagellates in the family of Symbiodiniaceae^6^ form critical symbiotic relationships with marine invertebrates, notably reef-building corals. Disruption of this symbiosis due to environmental stress can lead to bleaching and eventual death of the host animal. A few dinoflagellate lineages also form symbiotic relationships with zooplankton (*Brandtodinium nutricula*) and foraminifera (*Pelagodinium beii*). Comparison of annotated Pfam domains in these symbiotic taxa against those in all taxa, shows that functions related to protein-protein interaction (potentially involved in host-symbiont recognition^41–43^), extracellular matrix, photosynthesis, signal transduction, membrane transport, and cell adhesion are over-represented in the symbiotic lineages (Supplementary Table S9).

Earlier studies of Symbiodiniaceae genomes revealed extensive lineage-specific divergence^12–14,16^, and genome-wide positive selection of symbiosis-related functions^15^. Features known to be prevalent in Symbiodiniaceae, including *Chlorophyll a-b binding protein* (PF00504), *Ankyrin repeats (3 copies)* (PF12796) and *EF-hand domain pair* (PF13499)^12,14–16^, were also significantly over-represented. *Carbonic anhydrase* (PF00484), involved in carbon dioxide sequestration for photosynthesis, was likewise over-represented (Supplementary Table S9). Nitrogen has been shown to be important for dinoflagellate-coral symbiosis, particularly in nutrient-poor tropical waters. It has even been suggested that the coral host uses ammonium limitation as a means of controlling the symbiont population^44^. Terms for nitrogen utilisation (such as ammonium, nitrate, and nitrite transport) are over-represented, confirming the importance of these processes to symbiotic dinoflagellates. Analysis of available Symbiodiniaceae genomes has shown a high level of sequence divergence even between closely related lineages^15^.

### Core functions in cold-adapted dinoflagellates

Although most dinoflagellates occur in tropical and subtropical regions, a few psychrophilic species have been described. To identify the functional characteristics of cold-adapted dinoflagellates, we compared the four psychrophilic species (those isolated from either the Arctic or Antarctic circles): two Suessiales (*Polarella glacialis* CCMP1383 and *Polarella glacialis* CCMP2088) and two Peridiniales (*Heterocapsa arctica* CCMP445 and *Scrippsiella hangoei*-like SHHI-4) against all taxa. Pfam domains related to cold adaptation were over-represented (Supplementary Table S10). The *DUF3494* (PF11999) domain (which is shared by type 1 ice-binding proteins^45^) was the most significantly enriched, and *cold-shock* (PF00313) domain the third most enriched. *DUF347 (repeat of unknown function)* (PF03988) is the second most over-represented domain, *ATP synthase (E/31* *kDa) subunit* (PF01991) the fourth-most, and *Chlorophyll a-b-binding protein* (PF00504) the fifth-most. The enrichment of chlorophyll-binding proteins is likely due to the primarily photosynthetic lifestyle of cold-adapted dinoflagellates compared to the mixotrophic lifestyle of other dinoflagellate taxa.

We further compared the cold-adapted Peridiniales against all Peridiniales taxa (Supplementary Table S11). Mixotrophy was reported in *Scrippsiella* spp. and *Heterocapsa* spp^46^; they comprise six of the 11 Peridiniales taxa in our dataset. Over-represented domains in cold-adapted Peridiniales include *DUF347* (PF03988), *chlorophyll a-b-binding protein* (PF00504), *DUF3494* (PF11999), and *peridinin-chlorophyll a binding protein* (PF02429). Similarly, we compared the cold-adapted Suessiales against all members of this lineage (Supplementary Table S12) and did not observe a significant enrichment of domains related to photosynthetic functions. This observation may not be surprising, because Suessiales lineages are photoautotrophs. A large number of over-represented domains with functions related to RNA processing (e.g. *DEAD/DEAH box helicase* (PF00270) and multiple [PPR_2 and PPR_3] *PPR repeat* (PF01535) domains) were recovered in the cold-adapted Suessiales. The *Ion transport protein* (PF00520) domain is under-represented in these taxa (Supplementary Table S12).

Species that thrive in extreme cold conditions must adapt to slow enzyme kinetics, which results in a decreased rate of catalysis. One postulated mechanism to deal with this issue is the up-regulation of proteins or substrates that might otherwise limit biochemical processes. The quantity of synthesised ribosomal proteins^47^ and ATP^48^ has also been shown to increase with decreasing temperature in psychrophilic species. In cold-adapted dinoflagellates (Supplementary Table S10), a number of ATP synthase subunits, ribosomal proteins and photosynthesis-related domains are over-represented. Our results suggest that an increased genetic capacity for these functions in psychrophilic dinoflagellates may compensate for low enzyme kinetics. This hypothesis remains to be tested as additional genomic and functional data from these dinoflagellates become available.

## Conclusions

Our study represents the most comprehensive *in silico* analysis, to date, of dinoflagellate transcriptomes and their functional capacities. We offer the first glimpses into the inventory of dark proteins in dinoflagellates, highlighting putative functions. Dark proteins represent a treasure trove of knowledge into local adaptation, because their functions are directly related to the diversification of lineages. We also identify potential functions that are shared across all analyzed dinoflagellate datasets, thus representing a putative set of defining features for these taxa. Enrichment analysis identifies features that define selective constraints on dinoflagellates to toxin biosynthesis, and to symbiotic and cold-adapted lifestyles. These results provide a foundational platform for further investigations of lineage-specific diversification, and of adaptation of dinoflagellates to their environments. However, most dinoflagellate genes are known to be constitutively expressed irrespective of growth conditions^49,50^, thus these transcriptome datasets do not allow us to adequately assess niche-specific gene expression and functional features; these questions can be addressed when genome data from the relevant taxa become available. The development and deployment of genetic methods such as CRISPR-Cas9, transposon-based mutagenesis, and RNAi are urgently needed to test hypotheses about genes that putatively define locally adapted dinoflagellate lineages.

## Methods

### Data

The predicted protein sequences from 62 assembled transcriptomes were retrieved from the Microbial Eukaryote Transcriptome Sequencing Project (MMETSP)^18^. Transcriptomes of *Gambierdiscus caribaeus*^19^ and *Alexandrium tamarense* CCMP1598^20^ were also acquired to create the initial pool of transcriptomes used in this study (64 in total; Supplementary Table S1). Eight of these transcriptomes (*Akashiwo sanguinea* CCCM885, *Gyrodinium dominans* SPMC 103, *Lessardia elongata* SPMC 104, *Oxyrrhis marina* CCMP1788, *Prorocentrum lima* CCMP684, *Prorocentrum micans* CCCM845, *Pyrocystis lunula* CCCM517 and *Thoracosphaera heimii* CCCM670-CCMP1069) were removed because they contained <1000 proteins; *Crypthecodinium cohnii* Seligo and *Symbiodinium* sp. Clade A were also removed, as they are potentially mislabelled^22^.

The Benchmarking Universal Single-Copy Orthologs (BUSCO v3.0.2b)^26^ program (using the alveolate_stramenophiles_ensembl, eukaryota_odb9 and protists_ensembl datasets; retrieved 22 September 2017), BLASTP searches (v2.3.0, e-value 1e-10) using the same three BUSCO datasets and BLASTP searches (v2.3.0, e-value 1e-10) using the protein orthologs from the Core Eukaryotic Genes (CEGs)^21^ were used to assess the completeness of each transcriptome. Seven transcriptomes (*Alexandrium andersonii* CCMP2222, *Alexandrium fundyense* CCMP1719, *Alexandrium minutum* CCMP113, *Alexandrium tamarense* CCMP1598, *Amoebophrya* sp. Ameob2, *Oxyrrhis marina* CCMP1795, *Symbiodinium* [now *Fugacium*] *kawagutii* CCMP2468) which all had >80%, >40% and >65% missing genes in the alveolate-stramenophiles, eukaryota and protists datasets, and also had <80% recovery of CEGs, were removed.

The proportion of each transcriptome with similarity to the RefSeq bacterial proteins database (release 80) was assessed using BLASTP (v2.2.28, e-value 1e-10); sequences matching at >90% identity were considered as putative bacterial contaminants. All transcriptomes analysed had <1% of their sequences sharing >90% similarity with bacterial protein sequences; the highest proportion was found in *Glenodinium foliaceum* CCAP1116_3 (0.67%; 714 sequences) and *Symbiodinium* sp. D1a (now *Durusdinium*, 0.45%; 203 sequences). As the putative bacterial sequences in each transcriptome was <1%, all transcriptomes (including the putative bacterial sequences) were retained and no filtering was conducted. To reduce redundancy of protein sequences in each of the 47 transcriptome datasets, each dataset was clustered independently using CD-HIT (v4.6.5, identity 100%, word length 5)^51^; only the longest ‘representative’ sequences (Table 1) were retained and used in subsequent identification of homologous sets.

### Identification of homolog groups and phylogenetic reconstruction

Construction of a maximum-likelihood phylogenetic tree consisting of all samples used in this study was conducted using the method described in Price and Bhattacharya^22^. Putatively homologous protein sets were constructed using OrthoFinder v1.1.8 (inflation 1.5)^52^. Similar to the “set B” clusters in Price and Bhattacharya^22^, we selected sequence sets (represented by ≥ 10 taxa) in which all taxa have only one sequence representation except for one taxon *X* that has two copies. The two sequences from taxon *X* were then removed from the sequence set before phylogenetic inference. This approach yielded 1043 single-copy sets for phylogenetic inference. For each of these sets, the sequences were aligned using MAFFT v7.310^53^ (--localpair --maxiterate 1000). Alignments were trimmed in two stages using trimAL v1.2rev59^54^: (1) the automated heuristic selection method (-automated1) was first used, then (2) taxa in which 50% of the sequence did not overlap with 50% of the other sequences were removed (-resoverlap 0.5 -seqoverlap 50). A maximum-likelihood tree then was inferred using the partitioned analysis implemented in IQ-TREE v1.5.5^55^; the best evolutionary model for each trimmed alignment was selected using IQ-TREE^56^, with models considered unlinked. Support of nodes in the inferred consensus tree was determined using 2000 ultrafast bootstraps^24^. Alignment statistics were generated using AMAS^57^. The distance between our tree and the one published was calculated using the Robinson-Foulds metric as implemented in PHYLIP^58^.

### Functional annotation of proteins

Each protein was queried using BLASTp (v2.3.0; -evalue 1e-5, -max_target_seqs 20) against separate SwissProt and TrEMBL databases (UniProt release 2017_07). We consider a protein to be “dark” (without a known function) if it, or any protein in the set it is part of, has no significant match to any UniProt entry. Gene Ontology (GO; http://geneontology.org/) terms were assigned using UniProt-GOA mapping (release 2017_09). Membrane transporters were identified by linking SwissProt annotations (release 2016_06), assigned using BLASTp (v2.3.0; -evalue 1e-10, -max_target_seqs 20), with the transporter classifications present in the Transporter Classification Database (retrieved 26 May 2017)^30^. The transcriptomes were annotated with Pfam domains using pfam_scan.pl (v1.5; database release 30) at E-value < 0.001 following earlier studies^16,59,60^, and InterProScan (v5.27-66.0) using all analysis packages except SignalP. Proteins were considered to contain a putative transmembrane domain if identified as such by both the Phobius and TMHMM packages.

### Enrichment analysis of function

For Pfam domains and transporter classifications, each identifier was assessed for enrichment against a background set using Fisher’s exact test, with correction for multiple testing using the Benjamini and Hochberg method^61^. GO enrichment was conducted using the topGO R package^62^, applying the Fisher’s Exact test with the ‘elimination’ methods to correct for the hierarchical structure of GO terms.

### Comparison of amino acid profiles between dark versus annotated proteins

We performed a random subsampling test to assess the statistical significance of the difference in proportion we observed for each amino acid between the high-confidence dark and the annotated protein sets. In the subsampling step, for each amino acid, we sampled its proportion from 100 randomly selected individual sequences (in the annotated set versus the dark set), and conducted Student’s *t*-test to assess the significance of the difference between their means; a Benjamini-Hochberg^61^ adjusted *p*-value ≤ 0.05 is considered statistically significant. We carried out this subsampling step 10,000 times, and assessed the number of times that the difference in proportions (of each amino acid in turn) is significant between the two sets. At 95% confidence interval (≥ 9500 tests returned a significant adjusted *p*-value), the difference in proportions of the amino acid is considered significant.

## Electronic supplementary material

Supplementary Information and Figures

Supplementary Tables

## Data Availability

The sources of datasets analysed during the current study are included in this published article and its Supplementary Information files, as detailed in Supplementary Table S1.
