# Supplementary material for: Core genes in diverse dinoflagellate lineages include a wealth of conserved dark genes with unknown functions
Source: Sci Rep. 2018 Nov 21;8:17175. doi: 10.1038/s41598-018-35620-z (PMC6249206; doi:10.1038/s41598-018-35620-z)
Supplement: Supplementary file 1 — Supplementary Information and Figures [file 41598_2018_35620_MOESM1_ESM.pdf]

Supplementary Information

Core genes in diverse dinoflagellate lineages include a wealth of conserved dark genes with unknown functions

Timothy G. Stephens[1], Mark A. Ragan[1], Debashish Bhattacharya[2] and Cheong Xin Chan[1,3,*]

[1]Institute for Molecular Bioscience, The University of Queensland, Brisbane, QLD 4072, Australia

[2]Department of Biochemistry and Microbiology, Rutgers University, New Brunswick, NJ 08901, U.S.A.

[3]School of Chemistry and Molecular Biosciences, The University of Queensland, Brisbane, QLD 4072, Australia

*Corresponding author (c.chan1@uq.edu.au)

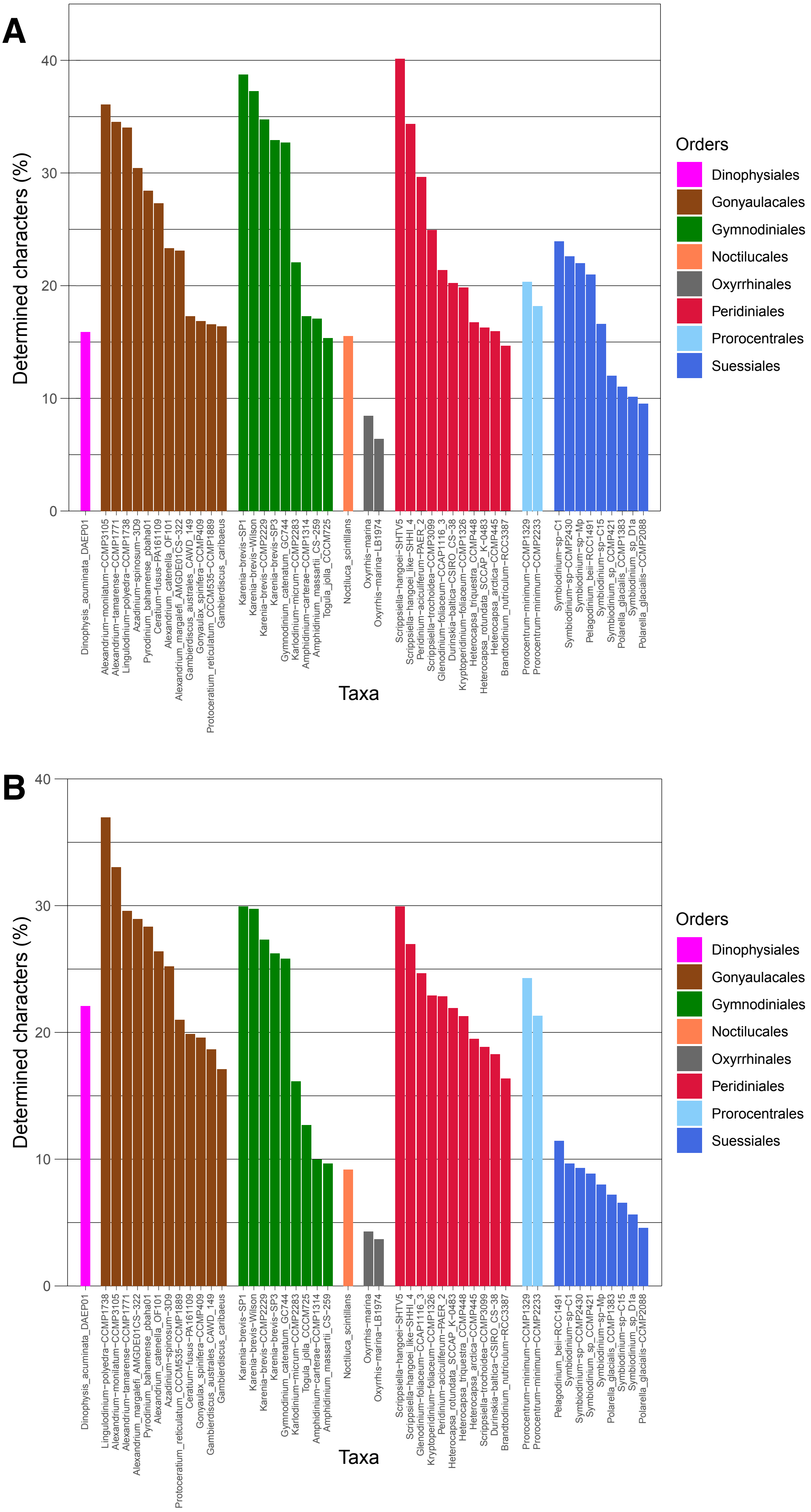

Supplementary Figure S1. Percentage of determined characters (aligned residues) for each taxon in the concatenated alignments used to construct (A) the reference dinoflagellate tree in Figure 1a and (B) the dark protein tree in Figure 3.

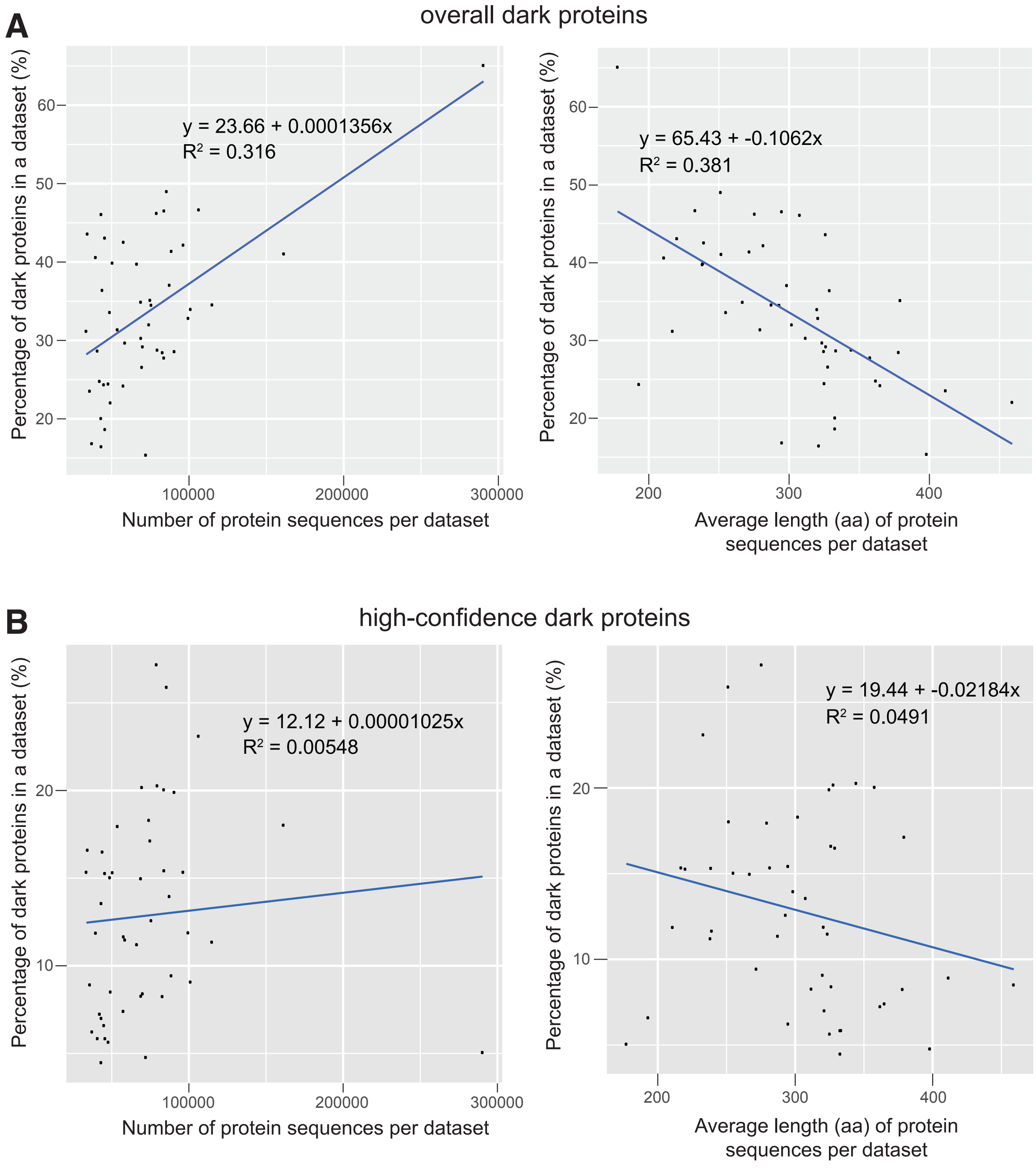

Supplementary Figure S2. Dark proteins in dinoflagellates. For (A) overall dark proteins and (B) high-confidence dark proteins, the scatterplot comparing the total number of proteins against the percentage of dark proteins in each dataset is shown on the left; and the scatterplot comparing the average length of protein sequences against the percentage of dark proteins in each dataset is shown on the right. All plots are shown with the trend line of linear regression.

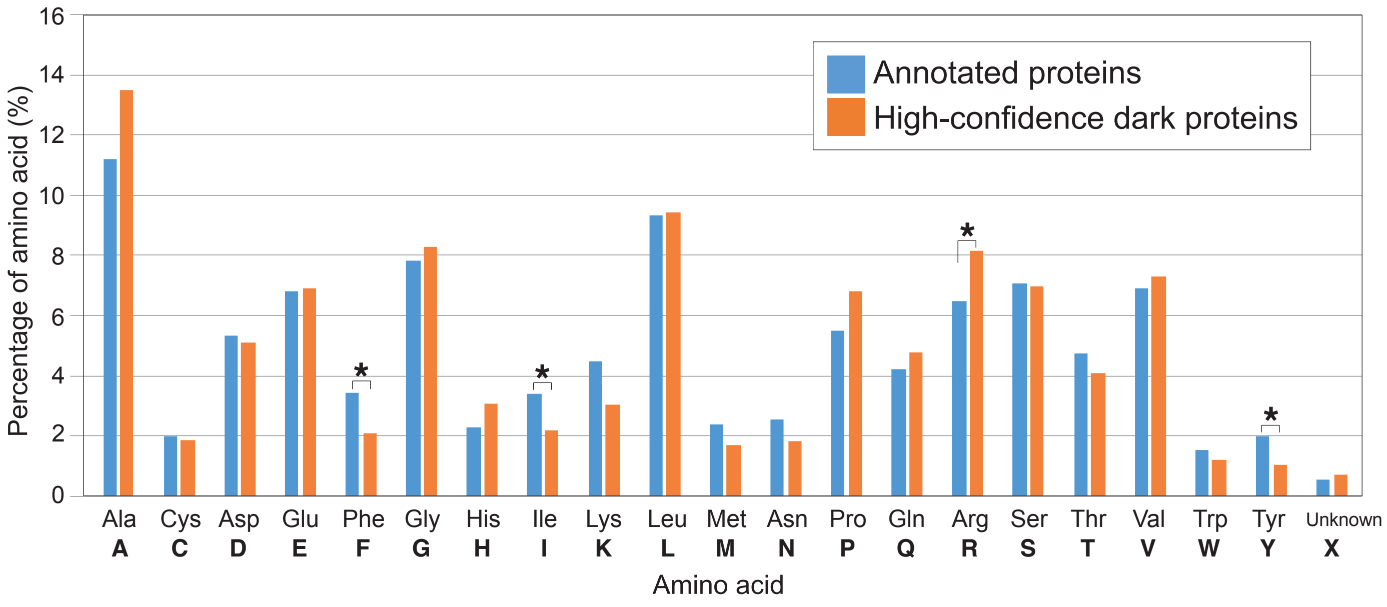

Supplementary Figure S3. Amino acid profiles of annotated proteins versus high-confidence dark proteins. Amino acids that are significantly different between the two sets, at 95% confidence interval of 10,000 Student's *t*-tests ($p \leq 0.05$; two-sided) comparing randomly subsampled 100 proteins from each set, are denoted with an asterisk (*).

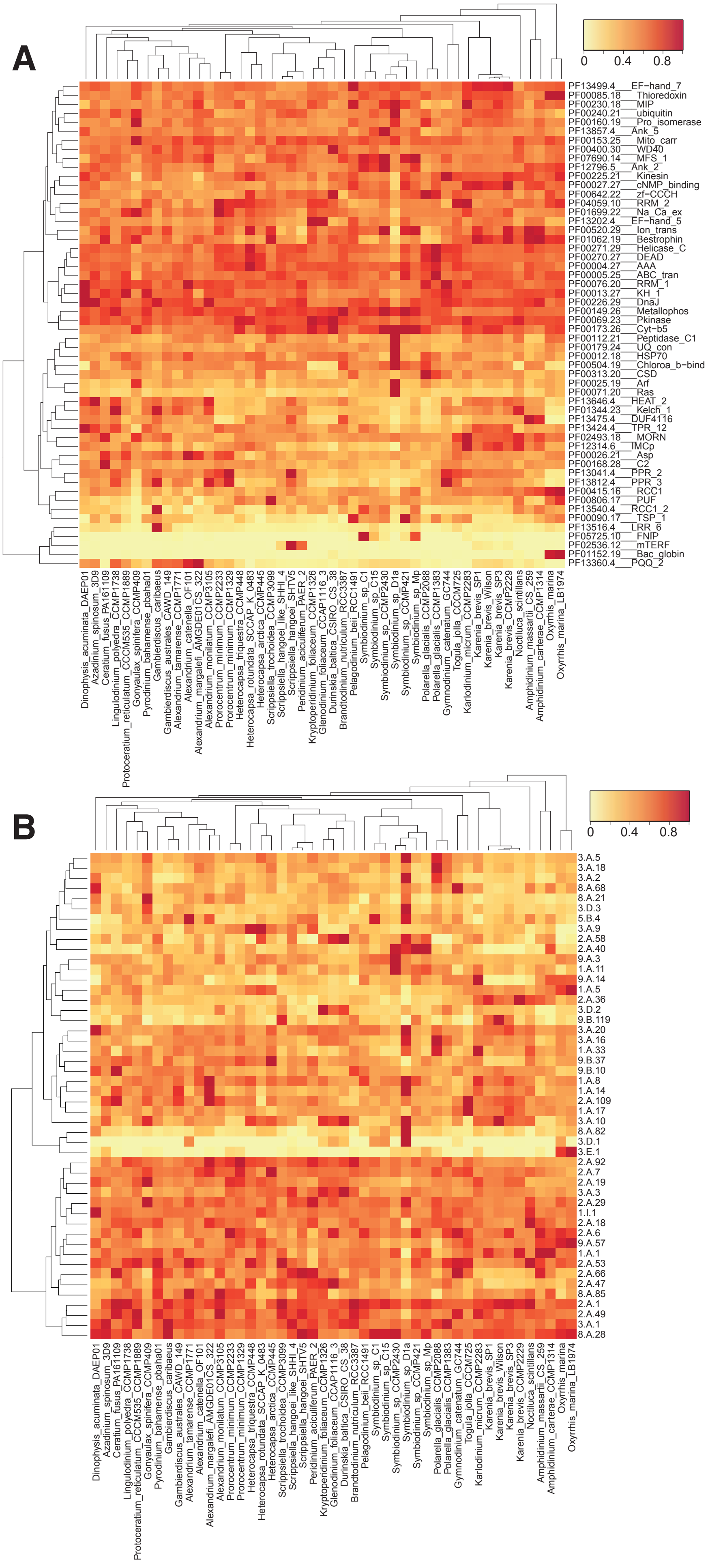

Supplementary Figure S4. Functional annotation of dinoflagellate genes. Heat map showing the abundance of (A) Pfam domains and (B) membrane transporters in the 47 taxa, showing the non-redundant top 20 most-abundant item in each taxon. The number per row (taxon) is normalised by its maximum value of that row.
